# RETRACTION: Kinesin Family Member 2A High Expression Correlates With Advanced Tumor Stages and Worse Prognosis in Non‐Small Cell Lung Cancer Patients

**DOI:** 10.1002/jcla.70236

**Published:** 2026-04-13

## Abstract

**RETRACTION**: G. Wang, Z. Wang, H. Yu, “Kinesin Family Member 2A High Expression Correlates With Advanced Tumor Stages and Worse Prognosis in Non‐Small Cell Lung Cancer Patients,” *Journal of Clinical and Laboratory Analysis* 34, no. 4 (2019): e23135, https://doi.org/10.1002/jcla.23135.

The above article, published online on 19 December 2019 in Wiley Online Library (wileyonlinelibrary.com), has been retracted by agreement between the journal Editor‐in‐Chief, Rong Fu; and Wiley Periodicals, LLC. The retraction has been agreed due to concerns raised by a third party, which identified compromised data and systematic issues in the published article. In addition, inappropriate image duplications were found in Figure 2 (panel (B)), which had been previously published by a different author group. The authors and their institutes were unresponsive when these concerns were brought to their attention. Accordingly, the article is retracted as the editors have lost trust in the reliability of the data and the results presented.

**RETRACTION**: G. Wang, Z. Wang, H. Yu, “Kinesin Family Member 2A High Expression Correlates With Advanced Tumor Stages and Worse Prognosis in Non‐Small Cell Lung Cancer Patients,” *Journal of Clinical and Laboratory Analysis* 34, no. 4 (2019): e23135, https://doi.org/10.1002/jcla.23135.

The above article, published online on 19 December 2019 in Wiley Online Library (wileyonlinelibrary.com), has been retracted by agreement between the journal Editor‐in‐Chief, Rong Fu; and Wiley Periodicals, LLC. The retraction has been agreed due to concerns raised by a third party, which identified compromised data and systematic issues in the published article. In addition, inappropriate image duplications were found in Figure 2 (panel (B)), which had been previously published by a different author group. The authors and their institutes were unresponsive when these concerns were brought to their attention. Accordingly, the article is retracted as the editors have lost trust in the reliability of the data and the results presented.

